# CNV-WebStore: Online CNV Analysis, Storage and Interpretation

**DOI:** 10.1186/1471-2105-12-4

**Published:** 2011-01-05

**Authors:** Geert Vandeweyer, Edwin Reyniers, Wim Wuyts, Liesbeth Rooms, R Frank Kooy

**Affiliations:** 1 Department of Medical Genetics, University Hospital Antwerp, Antwerp, Belgium; 2 University Hospital Antwerp, Antwerp, Belgium

## Abstract

**Background:**

Microarray technology allows the analysis of genomic aberrations at an ever increasing resolution, making functional interpretation of these vast amounts of data the main bottleneck in routine implementation of high resolution array platforms, and emphasising the need for a centralised and easy to use CNV data management and interpretation system.

**Results:**

We present CNV-WebStore, an online platform to streamline the processing and downstream interpretation of microarray data in a clinical context, tailored towards but not limited to the Illumina BeadArray platform. Provided analysis tools include CNV analsyis, parent of origin and uniparental disomy detection. Interpretation tools include data visualisation, gene prioritisation, automated PubMed searching, linking data to several genome browsers and annotation of CNVs based on several public databases. Finally a module is provided for uniform reporting of results.

**Conclusion:**

CNV-WebStore is able to present copy number data in an intuitive way to both lab technicians and clinicians, making it a useful tool in daily clinical practice.

## Background

Up to 12% of the humane genome is present in a variable number of copies, referred to as copy number variation (CNV) [1-3]. A small subset of specific CNVs is shown to associate with a wide spectrum of diseases [4-9].

Before the development of array-based comparative genomic hybridisation, visual inspection of karyotypes under the microscope limited the detection of chromosomal aberrations to events larger than 5 to 10 Mb. A major breakthrough was achieved with the generation of BAC-arrays, initially consisting of about 2000 large-insert clones spotted on a glass back plate [10]. With the capacity to screen a whole genome at once at a practical resolution of about 1.4 Mb, this type of array launched the CNV-analysis field in routine diagnostics. More recently, the resolution of BAC-arrays was improved using over 20000 tiling probes [2]. At present, BAC-arrays are gradually replaced by higher resolution platforms such as oligonucleotide- and SNP-arrays [11,12]. The major advantage of SNP-arrays over oligonucleotide- and BAC-arrays, is that they provide genotype information in addition to intensity ratios. Combining both information layers gives these platforms the potential to detect CNVs at a significantly higher resolution than the first generation platforms [13,14].

**Figure 1:**
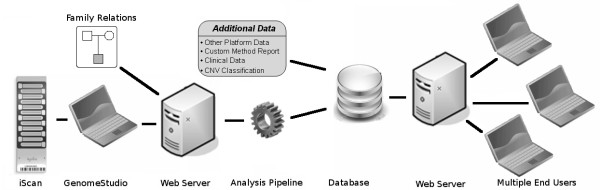
**Platform structure overview of CNV-WebStore**.

This increased resolution, in combination with the wide variety of data analysis methods, causes a shift of focus from data generation to data interpretation. To interpret these calls in a clinical context several aspects typically need to be taken into account [8] and several tools are available to aid in this process [15-17]. While many tools exist for either data analysis or presentation and interpretation, few exist that efficiently combine both [18-23]. Here, we present CNV-WebStore, a web-based platform that couples data analysis with data storage, visualisation, several downstream analysis methods and reporting tools, to serve as a complete data to report pipeline (figure 1).

The presented platform incorporates a majority vote based analysis pipeline specific for Illumina BeadArray data, capable to detect CNVs with a lower limit of 3 consecutive probes, mosaicism and uniparental disomy. Though analysis of data generated on other platforms is not supported, CNV sets from any platform can be uploaded for downstream analysis. This enables the integration of multiple experimental techniques in a single interpretation pipeline.

## Platform Implementation

### Interface architecture

CNV-WebStore is hosted on an 8 core, 64bit Suse Linux Enterprise Enterprise Server with 16 Gb RAM, running Apache v2.2.0, PHP v5.2.3 and MySQL v5.1.39. All interface components are composed of PHP and JavaScript, with support for most major browsers. Annotation data is automatically updated on a weekly basis using scheduled Perl scripts. Default data analysis times range from 2 minutes per sample for 300 K arrays, to 10 minutes for 1 M arrays.

### Data analysis packages

All analysis methods were run in their native programming language in a 64bit Linux environment, and combined by a wrapper script written in Perl v5.8.8 with multithreading support.

*QuantiSNP *v1.1 was installed and used confirming to the Academic licence. Parameters were set mainly at default values, namely 10 Expectation-Maximisation steps, 2 M as characteristic length for CNVs and a maximal copy number of 4. Additionally, correction for local GC content was applied. Minimal Log Bayes Factor was set at 8.5 based on experimental experience [24].

*PennCNV *rev081119 was installed and run with the generic markov model and population frequency of B-alleles, since no chip-specific files were available. No family information was used and minimal confidence was set to 10 based on experimental experience [25].

*VanillaICE *v1.4.0 was installed under R v2.9.0 and configured according to the authors suggestions for analysing Illumina data. Expected intensity ratios were adapted following technical guidelines from Illumina and emission probability calculations were set to cope with non-polymorphic probes. Samples were analysed individually, so we chose a robust in sample estimate of intensity variability as proposed by the author [26]. A complete overview of the used settings is available as additional file 1.

*BAFsegmentation *v1.1.0 was installed under R v2.9.0. Program parameters were set to 0.97 as 'informative_treshold', 0.8 as 'triplet_treshold', 0.56 as 'ai_treshold' and 4 as 'ai_size' [27].

*SNPtrio *[28] calculations were implemented as described by the authors using Perl.

## Results

### Data analysis approach

**Figure 2:**
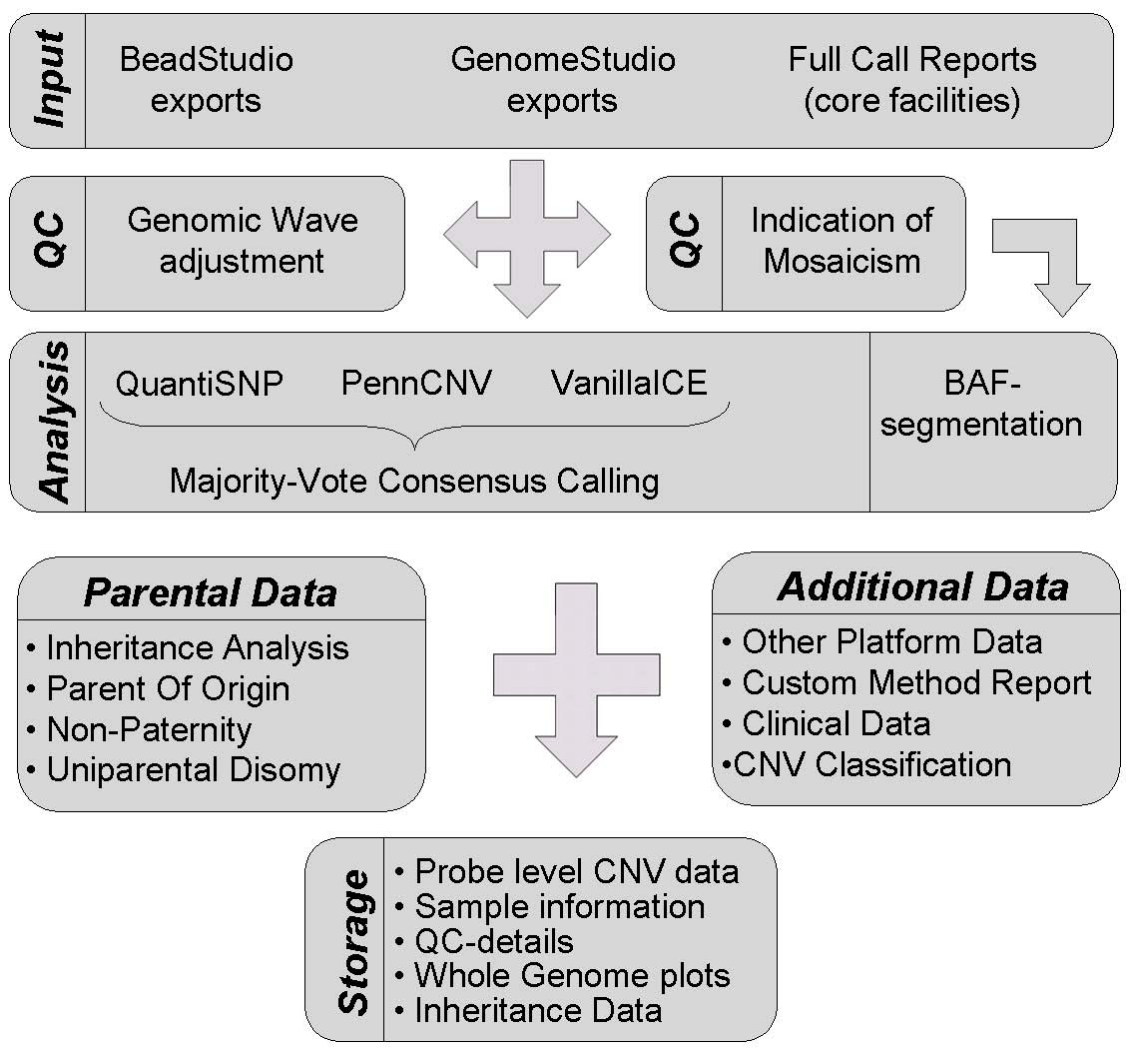
**Analysis pipeline implemented in CNV-WebStore for Illumina BeadArray data**.

To make a correct functional interpretation of CNV data, it is necessary to be able to start from a reliable set of candidate regions. We therefore implemented an analysis pipeline for the Illumina BeadArray platform, aimed at detecting rare, small scale CNVs. Since many methods have already been published and mutually compared for the analysis of SNP-array data, we decided to use existing methods, instead of creating yet another [24-26,29-31]. The pipeline uses the widely used approach of majority vote based calling, as previously described for the Affymetrix platform [32], to generate a restrictive set of results, with a higher sensitivity and specificity (figure 2). Specificity is increased by discarding CNVs called by only 1 of the 3 methods, usually representing method specific artefacts. An increase in sensitivity over each single method is reached by adding regions that were called by 2 methods, but not by the third. CNV delineation is done with a conservative overlap, retaining only regions seen by more than one method.

Previous studies comparing multiple computational methods for microarray analysis showed that Hidden Markov Model (HMM) based methods perform best in detecting rare and small CNVs. These methods explicitly combine intensity and genotype data, take the non-uniform probe spacing into account and have an underlying assumption of general diploidy, although they have a significantly larger computational footprint than classic aCGH segmentation methods [13,26,33]. In addition, results are sample size independent, in contrast to some recent methods that use a joint calling algorithm over multiple samples [34]. Although joint-calling can be a very powerful tool in GWA projects for the detection of recurrently variable regions, population dependent CNV calls are unacceptable in a clinical context where all results have to be reproducible under any circumstance. We chose QuantiSNP and PennCNV because they are the two most widely accepted and used HMM methods for Illumina data analysis today, and VanillaICE for its claimed high sensitivity to deletions, which are responsible for approximately 70% of pathogenic events described so far [8].

Besides general copy-number analysis, the data are screened for indications of mosaicism based on quality control parameters, as described below. BAFsegmentation is used with default settings to detect regions of aberrant B-allele frequency, and to estimate the proportion of cells containing the allelic imbalance [27].

Finally, uniparental disomy detection is implemented using the method described by Ting *et al*. [28]. Based on informative combinations of mendelian inconsistencies, uniparental heterodisomy, isodisomy and non-paternity can be deduced from the provided genotype data.

### Performance estimation

To estimate performance of the proposed majority voting, its stringency and sensitivity were compared against the separate methods using a reference dataset generated by Pinto *et al*. using an approach similar to ours [32], and a dataset of 192 HapMap samples generated by Illumina on the HumanCNV370-Quad BeadChip, from which 181 samples were also present in the former study. None of the separate methods performed significantly better in picking up the previously published CNV regions from the Illumina data (Table 1). Similar results were obtained using a set of CNVs seen in a study by McCarroll et al on the Affymetrix 6.0 array [3] (data not shown).

**Table 1:** Proportion of CNVs called by Pinto et al. detected by the different methods used

	Pinto *et al*.					
	**Deletions ***(n = 378)*		**Duplications ***(n = 130)*		**Overall ***(n = 508)*	

**Algorithm**	**%**	**P-Val**	**%**	**P-Val**	**%**	**P-Val**

2/3 Vote	81.5		69.6		74.4	
QuantiSNP	79.0	0.19	66.0	0.26	71.3	0.13
PennCNV	80.0	0.3	75.2	0.16	77.2	0.15
VanillaICE	83.0	0.31	55.1	0.0073	66.3	0.0023

Results are averaged over 181 HapMap samples. P-values are calculated using a one-tailed z-test for difference between proportions, comparing the majority vote against each separate method.

The slight sacrifice in sensitivity towards duplications, caused by incorporation of VanillaICE in the majority vote, can be countered by taking advantage of the observed high power of PennCNV in detecting duplication events. To do so, an option for asymmetric voting, where PennCNV duplication calls do not need to be confirmed by a second method, is available.

**Figure 3:**
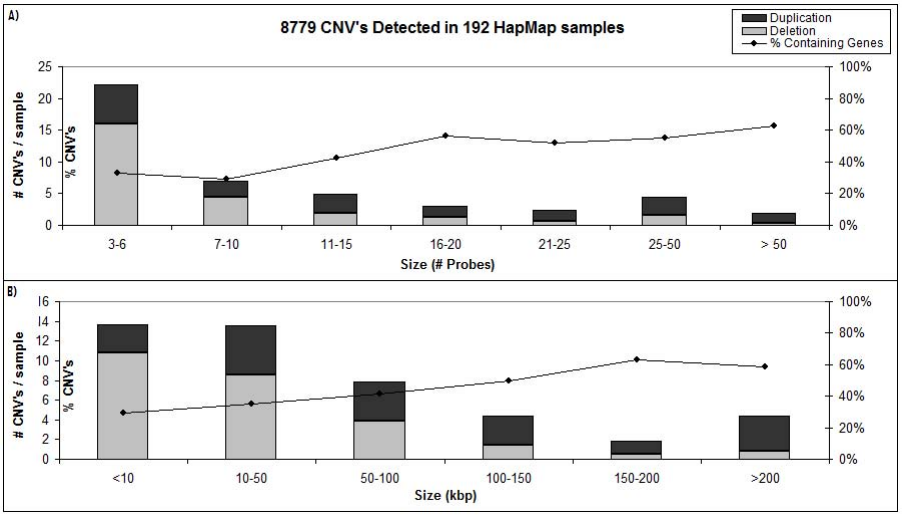
**Overview of 8779 copy number variations detected in 192 HapMap Samples**. On the left axis, the number of deletions per sample are shown as light bars, duplications as dark bars. On the right axis the percentage of aberrations in the bin, containing genes is shown. A) Classified by coverage. B) Classified by aberration size.

When applied to the full panel of 192 high quality HapMap samples, we retrieved a set of 8779 aberrations. This corresponds to a mean of 45 aberrations per sample and to 4% of the genome present in a variable number of copies over all the samples. This high number of CNVs per sample seems out of range with previous studies. When classified by size or coverage (figure 3), it is clear that most of the called CNVs are found in the category of 3-6 consecutive probes. Thus, it appears that the increase in resolution results in an increase in the number of additional small CNVs detected. Indeed, using an artificial resolution of 100 Kb, corresponding to 15 consecutive probes, we detected 8 CNVs per sample, in range with an average of 6 CNVs per sample detected on a 85 K oligonucleotide platform with a practical resolution of 100 Kb [4].

### Quality Control

Quality control parameters are stored during analysis and user feedback is given if they exceed preset values. High quality samples are considered to have a call rate of >99.4%, LogR standard Deviation < 0.2, B-allele Standard Deviation < 0.6 and absolute genomic wave < 0.03. In case of genomic waving, an event where the intensity data shows typical fluctuation across the chromosomes, normalisation is performed during CNV analysis [35]. QC-values are taken from PennCNV output.

Chromosome specific B-Allele standard deviation, calculated by QuantiSNP, is screened for significant outliers, as these might be an indication of mosaicism. When mosaicism is suspected, the user is informed of possible mosaicism and BAFsegmentation is started [27].

### Other Platform Data

To import generic CNV reports, custom parsers can be generated for any tab-separated file. Once imported into the database, family relations and raw probe level data can be added. When this raw data is available, inheritance examination and parent of origin analysis can be carried out as described below.

### Sample Annotation

Default annotation of samples consists of quality control data, used platform type, gender and the total number of detected aberrations. Additionally, clinical data can be added. Structured clinical information, conforming to the London neurogenetics nomenclature [36], aids in unambiguous comparison and grouping of samples based on their phenotype.

### CNV Annotation

**Figure 4:**
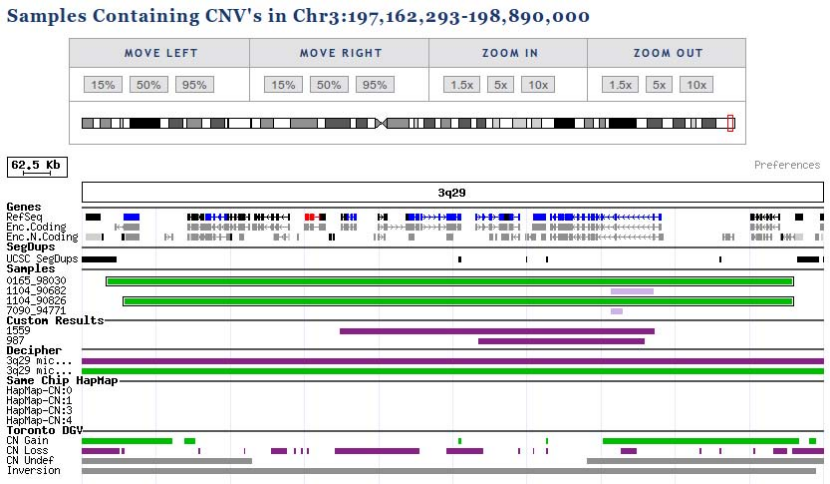
**Graphical representation of CNV-annotation by CNV-WebStore**. General color codes are green for duplication and purple for deletion events. OMIM information for genes is indicated by blue or red (MORBID) color.

Clinical interpretation of CNVs requires a comprehensive cross-linking of available annotation data. Besides experimental data such as aberration size, copy number state, inheritance data and genome build, several additional annotations are automatically added (figure 4).

Affected RefSeq genes [37] are listed, with associations to phenotypes extracted from OMIM [38]. Known disease related loci and published case reports are extracted from the DECIPHER and ECARUCA databases [15,39]. Finally, information on flanking segmental duplications is available, as these often flank recurrent microdeletion and -duplication regions [40]. The Toronto Database of Genomic Variants is a major collection of known benign variants detected in control populations [1]. The database comprises a total of almost 40 studies, from which a user can select a subset used for automatic annotation. As a second source of healthy controls, all HapMap samples analysed by Illumina to create the reference cluster files, passing our quality control requirements, were analysed by the described pipeline for all supported chip types and presented to the user as a chip-specific control set [11].

Based on supplied family information, CNVs are further annotated with inheritance information, parent of origin information for *de novo *events and uniparental iso- or heterodisomy prediction [28].

### Data management and protection

All data are stored in a relational database which is backed up on a daily basis. Access to specific data using CNV-WebStore is regulated by user-level permissions. Users are allowed to manage and share their own data with different users or user groups, with varying privileges, ranging from read-only access, over the privilege to only edit clinical or CNV annotations, to the right to further share data with more users. In order to guarantee data integrity, all changes in annotation are kept by a logging system. Sample anonymity is ensured as there is no storage of personal information, such as names or birth dates. Sample identification relies on arbitrary identification codes specified by the user. A typical example would be a numeric identifier linking to the DNA sample that was analysed.

### Data Representation

**Figure 5:**
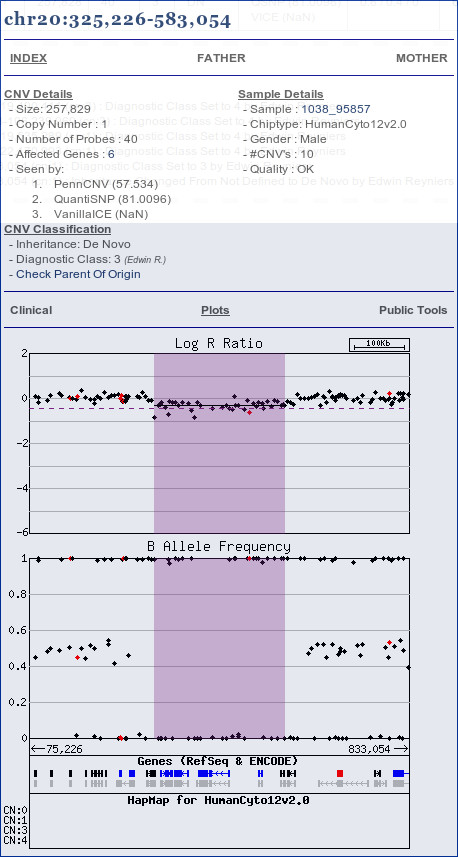
**Context Information presentation**. A single tooltip allows access to parental data (on top), clinical information and public resources (middle) and visual examination plots (bottom).

Centralised storage of all data allows flexible presentation and searching approaches. By default CNV-WebStore presents data in a compact graphical browser (see figure 4). CNV regions are denoted by bars using a color code representing copy number and clinical significance. Results from multiple experiments can be combined in a single overview or can be visualised separately. The graphical representation allows intuitive correlation of results with previous results, gene content, known pathogenic regions and benign variants. It further provides access to inheritance information, probe level data for visual examination, quality control results, general sample information and several public resources for extensive investigation of annotation data [38,41,42] (see figure 5). Individual users can select the resources they consider relevant to be included, or add new resources available to all users.

Alternatively, data for a single sample can also be presented in a tabular format. Here, clinical data is bundled with experimental data, CNV annotation, occurrence statistics and reporting tools. Additional annotation tools present from here are a direct PubMed querying tool to intersect the clinical data with gene content, and a CNV prioritization option based on the Endeavour program [19].

### Reporting

The reporting module of CNV-WebStore provides uniform and comprehensive summaries of annotated data. Users have the option to include clinical and experimental details, a schematic karyogram, ISCN representation [43], all or a filtered subset of detected aberrations, gene content and annotation history.

### Exporting

To analyse data using other tools, results can be exported as Tab separated files, BED files compatible with UCSC and Ensembl genome browsers, or XML files compatible with Illumina Genome Viewer.

## Discussion

SNP-based microarray platforms are used more and more in daily diagnostics, providing the potential to analyse a patient's genome at high resolution on both genotype and copy number level. As for many of today's technologies, the amount of produced data is vast and interpretation becomes a cumbersome task.

To overcome this, and to help sieve through the many generated CNV calls, we developed CNV-WebStore, a centralised analysis and interpretation platform, able to go from raw data to reports from a single interface. It implements a majority vote analysis pipeline combining three HMM based methods, each using slightly different underlying models, with a net effect of filtering out model specific artefacts. A similar approach has been successfully applied on Affymetrix 500 K SNP array data, to obtain a stringent CNV set consisting of the most reliable calls [32].

From our results, two observations were made underscoring that obtaining the correct functional correlation between genotype and phenotype is the major hurdle in routine microarray usage. First, about 30% of the smallest detectable CNVs on the used platform disrupt 1 or more known coding sequences, often affecting exonic sequence. Second, CNVs affecting only intronic sequence might still contribute to specific phenotypes, as intronic sequences often harbour non-coding transcripts, which are currently emerging as key regulators in many diseases [44-46]. Furthermore, we expect that with the rising probe density of current and future microarray platforms, and with new techniques such as next generation sequencing based CNV calling, the amount of small variants detected will keep increasing, stressing the importance of a straightforward interpretation platform such as CNV-WebStore.

Interpretation tools made available to the user are presented in a unified way, offering maximal information with minimal effort. CNV-WebStore offers both molecular data, automatic CNV annotation and clinical information, in the context of public reference data or previous experimental results. The option to correlate clinical information with extended annotation data from a single view is a feature seen in some public platforms such as DECIPHER, but lacking in other platforms for routine usage [15,21-23]. Furthermore, the option to analyse data from the platform itself, without the need to use a command line interface is a major benefit of our platform over existing, similar interpretation solutions [15,21]. Finally, the capability to infer parent of origin and uniparental disomy from stored genotyping data is a unique feature of CNV-WebStore with clear impact on functional interpretation of imprinting related disorders [47]. A drawback might be that HMM based methods assume integer copy number values, limiting the possible application of the presented analysis pipeline in cancer research [27]. On the other hand, as incorporation of post-analysis results is supported for any type of data, including better suited segmentation based methods, the platform can create a substantial added value for interpretation of these data.

By integrating both data analysis, storage and presentation into a single platform, the problem of computational resources might impose a practical limit on usability. To address this, a system monitor is implemented in the analysis pipeline that distributes the system load to those processors that are spare in the system. Cloud computing, as illustrated recently for the Reciprocal Smallest Distance Algorithm, was also considered [48]. As a hosting server with 8 cores and 2 Gb RAM per core is able to analyse samples with 1 M markers in less than 10 minutes, the actual limit lies in data storage and not in computational power, which is nowadays easily managed, without the large financial overhead of renting cloud-based storage.

## Conclusions

By combining microarray data analysis with centralised storage, a comprehensive start point was created for data interpretation, the major task in current diagnostic usage of microarray data. The ability to compare results against clinical information, previously analysed samples, known variant regions and gene content allows a sensitive selection of CNVs to obtain the most relevant results for further research. Extensive logging enables the tracking of changes to every sample or region, making CNV-WebStore a useful tool in a collaborative setup.

In conclusion, we have developed a web based platform providing an intuitive pipeline to go with minimal effort from raw data to functional interpretation and reporting of results. Because both lab technicians and clinical staff can annotate the data from their own expertise, the most relevant regions will most likely come forward. This makes CNV-WebStore a valuable tool in daily clinical practice, where modern techniques often produce overwhelming amounts of data.

## Availability and requirements

• Project Name: CNV-WebStore

• Project Home Page: http://sourceforge.net/projects/cnv-webstore

• WebService Address: http://medgen.ua.ac.be/cnv

• Operating Systems: UNIX-like 64bit OS

• Programming Language: Perl/PHP/R/MySQL

• Other Requirements: LAMP server

• License: GNU GPL, free for academic usage

## Authors' contributions

GV developed the software platform described and wrote the manuscript. ER and LR performed validation studies and contributed to the conceptual design of the platform. WW contributed to the conceptual design of the platform. RFK supervised the project and the final manuscript. All authors read and approved the final manuscript.

## Supplementary Material

Additional file 1 **VanillaICE code and settings**. VanillaICE settings were adapted to optimise performance on Illumina BeadChip data. This files contains the resulting R-code that can be used with version 1.4.0. Click here for file

## References

[B1] Iafrate AJ Feuk L Rivera MN Listewnik ML Donahoe PK Qi Y Scherer SW Lee C Detection of large-scale variation in the human genome Nat Genet 2004 36 9 949 951 10.1038/ng1416 15286789

[B2] Redon R Ishikawa S Fitch KR Feuk L Perry GH Andrews TD Fiegler H Shapero MH Carson AR Chen W Global variation in copy number in the human genome Nature 2006 444 7118 444 454 10.1038/nature05329 17122850 PMC2669898

[B3] McCarroll SA Kuruvilla FG Korn JM Cawley S Nemesh J Wysoker A Shapero MH de Bakker PI Maller JB Kirby A Integrated detection and population-genetic analysis of SNPs and copy number variation Nat Genet 2008 40 10 1166 1174 10.1038/ng.238 18776908

[B4] Walsh T McClellan JM McCarthy SE Addington AM Pierce SB Cooper GM Nord AS Kusenda M Malhotra D Bhandari A Rare structural variants disrupt multiple genes in neurodevelopmental pathways in schizophrenia Science (New York, NY 2008 320 5875 539 543 10.1126/science.1155174 18369103

[B5] Yoo SM Choi JH Lee SY Yoo NC Applications of DNA microarray in disease diagnostics Journal of microbiology and biotechnology 2009 19 7 635 742 19652509

[B6] Marshall CR Noor A Vincent JB Lionel AC Feuk L Skaug J Shago M Moessner R Pinto D Ren Y Structural variation of chromosomes in autism spectrum disorder Am J Hum Genet 2008 82 2 477 488 10.1016/j.ajhg.2007.12.009 18252227 PMC2426913

[B7] Lanktree M Hegele RA Copy number variation in metabolic phenotypes Cytogenet Genome Res 2008 123 1-4 169 175 10.1159/000184705 19287152

[B8] Koolen DA Pfundt R de Leeuw N Hehir-Kwa JY Nillesen WM Neefs I Scheltinga I Sistermans E Smeets D Brunner HG Genomic microarrays in mental retardation: a practical workflow for diagnostic applications Hum Mutat 2009 30 3 283 292 10.1002/humu.20883 19085936

[B9] McMullan DJ Bonin M Hehir-Kwa JY de Vries BBA Dufke A Rattenberry E Steehouwer M Moruz L Pfundt R de Leeuw N Molecular karyotyping of patients with unexplained mental retardation by SNP arrays: a multicenter study Hum Mutat 2009 30 7 1082 1092 10.1002/humu.21015 19388127

[B10] Snijders AM Nowak N Segraves R Blackwood S Brown N Conroy J Hamilton G Hindle AK Huey B Kimura K Assembly of microarrays for genome-wide measurement of DNA copy number Nat Genet 2001 29 3 263 264 10.1038/ng754 11687795

[B11] Peiffer DA Le JM Steemers FJ Chang W Jenniges T Garcia F Haden K Li J Shaw CA Belmont J High-resolution genomic profiling of chromosomal aberrations using Infinium whole-genome genotyping Genome Res 2006 16 9 1136 1148 10.1101/gr.5402306 16899659 PMC1557768

[B12] Stankiewicz P Beaudet AL Use of array CGH in the evaluation of dysmorphology, malformations, developmental delay, and idiopathic mental retardation Curr Opin Genet Dev 2007 17 3 182 192 10.1016/j.gde.2007.04.009 17467974

[B13] Yu T Ye H Sun W Li KC Chen Z Jacobs S Bailey DK Wong DT Zhou X A forward-backward fragment assembling algorithm for the identification of genomic amplification and deletion breakpoints using high-density single nucleotide polymorphism (SNP) array BMC bioinformatics 2007 8 145 10.1186/1471-2105-8-145 17477871 PMC1868765

[B14] Hester SD Reid L Nowak N Jones WD Parker JS Knudtson K Ward W Tiesman J Denslow ND Comparison of comparative genomic hybridization technologies across microarray platforms J Biomol Tech 2009 20 2 135 151 19503625 PMC2685605

[B15] Firth HV Richards SM Bevan AP Clayton S Corpas M Rajan D Van Vooren S Moreau Y Pettett RM Carter NP DECIPHER: Database of Chromosomal Imbalance and Phenotype in Humans Using Ensembl Resources Am J Hum Genet 2009 84 4 524 533 10.1016/j.ajhg.2009.03.010 19344873 PMC2667985

[B16] Aerts S Lambrechts D Maity S Van Loo P Coessens B De Smet F Tranchevent L-C De Moor B Marynen P Hassan B Gene prioritization through genomic data fusion Nat Biotechnol 2006 24 5 537 544 10.1038/nbt1203 16680138

[B17] Qiao Y Harvard C Tyson C Liu X Fawcett C Pavlidis P Holden JJ Lewis ME Rajcan-Separovic E Outcome of array CGH analysis for 255 subjects with intellectual disability and search for candidate genes using bioinformatics Hum Genet 128 2 179 194 10.1007/s00439-010-0837-0 20512354

[B18] Lai WR Johnson MD Kucherlapati R Park PJ Comparative analysis of algorithms for identifying amplifications and deletions in array CGH data Bioinformatics (Oxford, England) 2005 21 19 3763 3770 10.1093/bioinformatics/bti611 16081473 PMC2819184

[B19] Tranchevent LC Barriot R Yu S Van Vooren S Van Loo P Coessens B De Moor B Aerts S Moreau Y ENDEAVOUR update: a web resource for gene prioritization in multiple species Nucleic Acids Res 2008 36 Web Server W377 384 10.1093/nar/gkn325 18508807 PMC2447805

[B20] Hehir-Kwa JY Wieskamp N Webber C Pfundt R Brunner HG Gilissen C de Vries BB Ponting CP Veltman JA Accurate distinction of pathogenic from benign CNVs in mental retardation PLoS computational biology 2010 6 4 e1000752 10.1371/journal.pcbi.1000752 20421931 PMC2858682

[B21] Gai X Perin JC Murphy K O'Hara R D'Arcy M Wenocur A Xie HM Rappaport EF Shaikh TH White PS CNV Workshop: an integrated platform for high-throughput copy number variation discovery and clinical diagnostics BMC bioinformatics 2010 11 74 10.1186/1471-2105-11-74 20132550 PMC2827374

[B22] Kim SY Nam SW Lee SH Park WS Yoo NJ Lee JY Chung YJ ArrayCyGHt: a web application for analysis and visualization of array-CGH data Bioinformatics (Oxford, England) 2005 21 10 2554 2555 10.1093/bioinformatics/bti357 15746288

[B23] Menten B Pattyn F De Preter K Robbrecht P Michels E Buysse K Mortier G De Paepe A van Vooren S Vermeesch J arrayCGHbase: an analysis platform for comparative genomic hybridization microarrays BMC bioinformatics 2005 6 124 10.1186/1471-2105-6-124 15910681 PMC1173083

[B24] Colella S Yau C Taylor JM Mirza G Butler H Clouston P Bassett AS Seller A Holmes CC Ragoussis J QuantiSNP: an Objective Bayes Hidden-Markov Model to detect and accurately map copy number variation using SNP genotyping data Nucleic Acids Res 2007 35 6 2013 2025 10.1093/nar/gkm076 17341461 PMC1874617

[B25] Wang K Li M Hadley D Liu R Glessner J Grant SF Hakonarson H Bucan M PennCNV: an integrated hidden Markov model designed for high-resolution copy number variation detection in whole-genome SNP genotyping data Genome Res 2007 17 11 1665 1674 10.1101/gr.6861907 17921354 PMC2045149

[B26] Scharpf RB Parmigiani G Pevsner J Ruczinski I Hidden Markov models for the assessment of chromosomal alterations using high-throughput SNP arrays The annals of applied statistics 2008 2 2 687 713 10.1214/07-AOAS155 19609370 PMC2710854

[B27] Staaf J Lindgren D Vallon-Christersson J Isaksson A Goransson H Juliusson G Rosenquist R Hoglund M Borg A Ringner M Segmentation-based detection of allelic imbalance and loss-of-heterozygosity in cancer cells using whole genome SNP arrays Genome Biol 2008 9 9 R136 10.1186/gb-2008-9-9-r136 18796136 PMC2592714

[B28] Ting JC Roberson ED Miller ND Lysholm-Bernacchi A Stephan DA Capone GT Ruczinski I Thomas GH Pevsner J Visualization of uniparental inheritance, Mendelian inconsistencies, deletions, and parent of origin effects in single nucleotide polymorphism trio data with SNPtrio Hum Mutat 2007 28 12 1225 1235 10.1002/humu.20583 17661425

[B29] Sun W Wright FA Tang Z Nordgard SH Loo PV Yu T Kristensen VN Perou CM Integrated study of copy number states and genotype calls using high-density SNP arrays Nucleic Acids Res 2009 19581427 10.1093/nar/gkp493 PMC2935461

[B30] Yavas G Koyuturk M Ozsoyoglu M Gould MP LaFramboise T An optimization framework for unsupervised identification of rare copy number variation from SNP array data Genome Biol 2009 10 10 R119 10.1186/gb-2009-10-10-r119 19849861 PMC2784334

[B31] Lin M Wei LJ Sellers WR Lieberfarb M Wong WH Li C dChipSNP: significance curve and clustering of SNP-array-based loss-of-heterozygosity data Bioinformatics (Oxford, England) 2004 20 8 1233 1240 10.1093/bioinformatics/bth069 14871870

[B32] Pinto D Marshall C Feuk L Scherer SW Copy-number variation in control population cohorts Hum Mol Genet 2007 16 Spec No 2 R168 173 10.1093/hmg/ddm241 17911159

[B33] Yau C Holmes CC CNV discovery using SNP genotyping arrays Cytogenet Genome Res 2008 123 1-4 307 312 10.1159/000184722 19287169

[B34] Alonso A Julia A Tortosa R Canaleta C Canete JD Ballina J Balsa A Tornero J Marsal S CNstream: A method for the identification and genotyping of copy number polymorphisms using Illumina microarrays BMC bioinformatics 2010 11 1 264 10.1186/1471-2105-11-264 20482829 PMC3098064

[B35] Diskin SJ Li M Hou C Yang S Glessner J Hakonarson H Bucan M Maris JM Wang K Adjustment of genomic waves in signal intensities from whole-genome SNP genotyping platforms Nucleic Acids Res 2008 36 19 e126 10.1093/nar/gkn556 18784189 PMC2577347

[B36] Fryns JP de Ravel TJ London Dysmorphology Database, London Neurogenetics Database and Dysmorphology Photo Library on CD-ROM [Version 3] 2001R. M. Winter, M. Baraitser, Oxford University Press, ISBN 019851-780, pound sterling 1595 Hum Genet 2002 111 1 113 10.1007/s00439-002-0759-6

[B37] Pruitt KD Tatusova T Klimke W Maglott DR NCBI Reference Sequences: current status, policy and new initiatives Nucleic Acids Res 2009 37 Database D32 36 10.1093/nar/gkn721 18927115 PMC2686572

[B38] Hamosh A Scott AF Amberger JS Bocchini CA McKusick VA Online Mendelian Inheritance in Man (OMIM), a knowledgebase of human genes and genetic disorders Nucleic Acids Res 2005 33 Database D514 517 15608251 10.1093/nar/gki033 PMC539987

[B39] Feenstra I Fang J Koolen DA Siezen A Evans C Winter RM Lees MM Riegel M de Vries BB Van Ravenswaaij CM European Cytogeneticists Association Register of Unbalanced Chromosome Aberrations (ECARUCA); an online database for rare chromosome abnormalities Eur J Med Genet 2006 49 4 279 291 10.1016/j.ejmg.2005.10.131 16829349

[B40] Lupski JR Stankiewicz P Genomic disorders: molecular mechanisms for rearrangements and conveyed phenotypes PLoS Genet 2005 1 6 e49 10.1371/journal.pgen.0010049 16444292 PMC1352149

[B41] Rebholz-Schuhmann D Kirsch H Arregui M Gaudan S Riethoven M Stoehr P EBIMed--text crunching to gather facts for proteins from Medline Bioinformatics (Oxford, England) 2007 23 2 e237 244 10.1093/bioinformatics/btl302 17237098

[B42] Hoffmann R A wiki for the life sciences where authorship matters Nat Genet 2008 40 9 1047 1051 10.1038/ng.f.217 18728691

[B43] Shaffer LGSM Campbell LJ ISCN 2009: An International System for Human Cytogenetic Nomenclature 2009 126

[B44] Hinske LC Galante PA Kuo WP Ohno-Machado L A potential role for intragenic miRNAs on their hosts' interactome BMC Genomics 2010 11 533 10.1186/1471-2164-11-533 20920310 PMC3091682

[B45] White NM Yousef GM MicroRNAs: exploring a new dimension in the pathogenesis of kidney cancer BMC Med 2010 8 1 65 10.1186/1741-7015-8-65 20964839 PMC2978114

[B46] Gatto S Ragione FD Cimmino A Strazzullo M Fabbri M Mutarelli M Ferraro L Weisz A D'Esposito M Matarazzo MR Epigenetic alteration of microRNAs in DNMT3B-mutated patients of ICF syndrome Epigenetics 2010 5 5 10.4161/epi.5.5.11999 20448464

[B47] Yamazawa K Ogata T Ferguson-Smith AC Uniparental disomy and human disease: an overview Am J Med Genet C Semin Med Genet 2010 154C 3 329 334 10.1002/ajmg.c.30270 20803655

[B48] Wall DP Kudtarkar P Fusaro VA Pivovarov R Patil P Tonellato PJ Cloud computing for comparative genomics BMC bioinformatics 2010 11 259 20482786 10.1186/1471-2105-11-259 PMC3098063

