# Supplementary material for: CNV-WebStore: Online CNV Analysis, Storage and Interpretation
Source: BMC Bioinformatics. 2011 Jan 5;12:4. doi: 10.1186/1471-2105-12-4 (PMC3024943; doi:10.1186/1471-2105-12-4)
Supplement: Additional file 1 — VanillaICE code and settings. VanillaICE settings were adapted to optimise performance on Illumina BeadChip data. This files contains the resulting R-code that can be used with version 1.4.0. [file 1471-2105-12-4-S1.DOC]

###################

# VanillaICE R script #

###################

# This script can only be used on single sample input files in format:

# probename TAB chr TAB position TAB logR TAB baf TAB genotype

# Load necessary packages & variables

message("loading libraries and functions")

library("VanillaICE")

library("genefilter")

library("SNPchip")

args <- commandArgs(TRUE)

datafile <- args[1]

gender <- args[2]

prefix <- args[3]

sampleID <- args[4]

taufactor <- args[5]

taufactor <- as.numeric(taufactor)

hmm <- args[6]

variance <- args[7]

chiptype <- args[8]

# Hidden markov model : experimental for Illumina data

if (hmm == "regular" ) {

mu <- log2(c(0, 1, 2, 2, 3, 4)/2)

}

if (hmm == "experimental") {

mu <- c(0, -0.45, 0, 0, 0.3, 0.75)

}

mu[1] <- log2(0.05/2)

# Load the list of non-polymorphic probes for the used chip

if (chiptype == "") {

zeroedfile = "zeroedsnps.txt"

}

if (chiptype != "") {

zeroedfile <- paste(chiptype, ".zeroed.txt", sep = "")

}

message("read in datafiles")

zeroed<- read.table(zeroedfile, as.is = TRUE, header = TRUE, sep = "\t")

zero<-zeroed$Name

message(paste("Loaded ",length(zero)," zero'ed probes.",sep=""))

# Read in sample data

sample <- read.table(datafile, as.is = TRUE, header = TRUE, sep = "\t")

nologR<-sample[sample[,grep("Log.R",colnames(sample))]=="NaN","Name"]

message(paste("Discarded ",length(nologR)," datapoints by lack of LogR value.",sep=""))

sample<-sample[!sample$Name %in% nologR,]

# Set Non-called Genotype to non-informative for non-polymorphic probes

sample[sample$Name %in% zero,grep(".GType", colnames(sample))] <- 5

# Convert pseudo-autosomal region to regular X

sample[sample$Chr == "XY","Chr"] <- "X"

# Convert genotypes to codes

GT <- sample[, grep(".GType", colnames(sample)), drop = FALSE]

GT[GT == "AA"] <- 1

GT[GT == "AB"] <- 2

GT[GT == "BB"] <- 3

GT[GT == "NC"] <- 4

GT <- as.matrix(as.integer(GT[[1]]))

# extract copy numbers

CN <- as.matrix(as.numeric(sample[, grep("Log.R.Ratio", colnames(sample))]))

colnames(GT) <- colnames(CN) <- sampleID

rownames(GT) <- rownames(CN) <- sample[, "Name"]

# Create feature data frame

fD <- new("AnnotatedDataFrame",

data = data.frame(position = sample[, "Position"],

chromosome = integer2chromosome(sample[, "Chr"]), stringsAsFactors=FALSE),

varMetadata = data.frame(labelDescription = c("position","chromosome")))

featureNames(fD) <- sample[, "Name"]

# Create oligoSnpSet object

myObject <- new("oligoSnpSet", copyNumber = CN,

calls = GT,

phenoData = annotatedDataFrameFrom(CN, byrow = FALSE),

featureData = fD, annotation = "Illumina")

# Sort data

message("Sorting data")

myObject <- myObject[order(chromosome(myObject), position(myObject)), ]

# Define the HMM

states <- c("homozygousDeletion", "hemizygousDeletion", "normal", "LOH",

"3copyAmp", "4copyAmp")

#probability of a homozygous genotype call

probs <- c(0.99, 0.9999, 0.99, 0.9999, 0.99, 0.99)

#Probability of a "Not-called"

probMissing <- c(0.999, rep(0.01, 5))

# Calculate robust estimate on standard devation (all but X)

message("Calculating Variation estimation")

NoXY <- sample[sample[,"Chr"]!="X",c("Name","Chr" )]

NoXY <- NoXY[NoXY[,"Chr"]!="Y","Name"]

sddata<-sample[sample$Name %in% NoXY,grep("Log.R",colnames(sample)) ]

message(paste("Variance model in use: ",variance, sep = ""))

if (variance == "insample") { # robust estimate in sample

robustSD <- function(X) (diff(quantile(X, probs=c(0.16, (1-0.16)), na.rm=TRUE))/2)[[1]]

uncertainty <- robustSD(sddata)

uncertainty <- matrix(uncertainty, nrow=nrow(myObject), ncol=ncol(myObject))

}

if (variance == "robustref") { # robust estimate as previous, from hapmap set (only for HumanCNV370-quad) !

robustSD <- read.table("robust.txt", as.is = TRUE, sep = "\t", header = TRUE)

uncertainty <- robustSD$Variance

uncertainty <- as.array(uncertainty)

rownames(uncertainty) <- robustSD$Probe

uncertainty <- as.matrix(uncertainty)

}

if (variance == "stdevref") { # st.dev from hapmap set (only for HumanCNV370-quad) !

stdev <- read.table("stdev.txt", as.is = TRUE, sep = "\t", header = TRUE)

uncertainty <- stdev$Variance

uncertainty <- as.array(uncertainty)

rownames(uncertainty) <- stdev$Probe

uncertainty <- as.matrix(uncertainty)

}

# Calculate intensity emission probabilities

logemission.logCT <- copynumberEmission(copynumber=copyNumber(myObject),

states=states,

mu=mu,

uncertainty=uncertainty,

takeLog=FALSE,

verbose=FALSE)

logemission.logCT[logemission.logCT < -10] <- -10

#load custom genotypeEmission Function (set p(NC | zeroed snp) = 1 )

source("genotypeEmission.R")

# Calculate genotype emission probabilities

names(probs) <- names(probMissing) <- states

GT <- calls(myObject)

logemission.gt <- genotypeEmission(genotypes = GT,

states = states,

probHomCall = probs,

probMissing = probMissing,

verbose = TRUE)

# Calculate complete emission probabilities

logemission <- logemission.gt + logemission.logCT

# Define a transition probability

tau <- exp(-2*diff(position(myObject))/(100*taufactor))

# define intitial state probabilities

initialStateProb <- rep(1e-04, length(states))

initialStateProb[states == "normal"] <- 1 - (length(states) - 1) * 1e-04

# specify the chromosomal arms (fits a separate HMM to each arm)

data(chromosomeAnnotation, package="SNPchip", envir=environment())

chrAnn <- as.matrix(chromosomeAnnotation)

chromosomeArm <- as.character(position(myObject) <= chromosomeAnnotation[chromosome(myObject), "centromereStart"])

chromosomeArm[chromosomeArm == "TRUE"] <- "p"

chromosomeArm[chromosomeArm == "FALSE"] <- "q"

# Apply viterbi fitting

message("Fitting the HMM")

fit <- viterbi(initialStateProbs=log(initialStateProb),

emission=logemission[, 1, ],

arm=chromosomeArm,

tau=tau,

)

# Search Breakpoints

message("Searching Breakpoints")

source("findBreaks.R")

results <- findBreaks(x = fit, states = states, position = position(myObject),

chromosome = chromosome(myObject), sample = sampleNames(myObject))

# only keep non-diploid regions

altered <- results[results$state != "normal", ]

altered[,"chr"] <- unlist(altered[,"chr"])

nrows <- nrow(altered)

filename <- paste("rawcnv/",prefix,".rawcnv",sep="")

headers <- c("Chr", "Start", "End", "Size", "StartProbe", "EndProbe", "NrSNPs", "State")

write(headers, file=filename, append = FALSE, sep = "\t", ncolumns = 8)

for(i in 1:nrows) {

startprobe <- sample[sample$Position == altered[i, "start"],"Name"]

endprobe <- sample[sample$Position == altered[i, "end"], "Name"]

line <- c(altered[i, "chr"][1], altered[i, "start"][1], altered[i, "end"][1],

altered[i, "nbases"][1], startprobe, endprobe, altered[i, "nprobes"][1],

altered[i, "state"][1])

write(line, file = filename, append = TRUE, sep= "\t", ncolumns = 8)

}

############################

# CUSTOM genotypEmission.R #

############################

genotypeEmission <- function (genotypes, states, probHomCall, probMissing, verbose = TRUE)

{

if (!is.numeric(genotypes))

stop("genotypes must be integers (1=AA, 2=AB, 3=BB, 4=missing, 5=zeroed")

emissionForGenotypes <- function(probHomGenotype, genotypes) {

isHom <- which(as.vector(genotypes) == 1 | as.vector(genotypes) == 3)

isHet <- which(as.vector(genotypes) == 2)

isMissing <- which(as.vector(genotypes) == 4 | is.na(as.vector(genotypes)))

# Include information for non-polymorphic probes

isZeroed <- which(as.vector(genotypes) == 5 )

emission.gt <- rep(NA, length(genotypes))

emission.gt[isHom] <- probHomGenotype

emission.gt[isHet] <- 1 - probHomGenotype

emission.gt[isMissing] <- NA

# Non-polymorphic probes have equal chance to represent each state

emission.gt[isZeroed] <- rep(1,length(probHomGenotype))

emission.gt

}

emission.gt <- array(NA, dim = c(nrow(GT), ncol(GT), length(states)))

for (j in 1:ncol(GT)) {

emission.gt[, j, ] <- sapply(probs, emissionForGenotypes, genotypes = GT[, j])

if (any(is.na(emission.gt[, j, 1]))) {

missing <- is.na(emission.gt[, j, 1])

if (!missing(probMissing)) {

if (length(probMissing) != length(states))

stop("probMissing must be a numeric vector equal

to the number of states")

emission.gt[missing, j, ] <- matrix(probMissing,

sum(missing), length(states), byrow = TRUE)

}

else {

if (verbose)

message("Argument probMissing is not specified.

Assume that missing genotype calls are independent of the underling hidden state")

emission.gt[missing, j, ] <- 1

}

}

}

dimnames(emission.gt) <- list(rownames(genotypes), colnames(genotypes), states)

return(suppressWarnings(log(emission.gt)))

}

#######################

# CUSTOM findBreaks.R #

#######################

findBreaks <- function(x, states, position, chromosome, sample,

lik1, lik2, chromosomeAnnotation){

if(is.matrix(x)) if(ncol(x) > 1) stop("x should be a vector or matrix with 1 column") if(!is.integer(chromosome)) {

chromosome <- chromosome2integer(chromosome)

}

if(!all(chromosome %in% 1:24)){

message("Chromosome annotation is currently available for

chromosomes 1-22, X and Y")

message("Please add/modify data(chromosomeAnnotation,

package='SNPchip') to accomodate special chromosomes")

stop()

}

if(!is.integer(position)) {

message("Coerced position to an integer.")

position <- as.integer(position)

}

##ensure that the reported breaks do not span the centromere

if(missing(chromosomeAnnotation)){

data(chromosomeAnnotation, package="SNPchip", envir=environment())

chrAnn <- as.matrix(chromosomeAnnotation)

}

chromosome <- integer2chromosome(chromosome)

uchrom <- unique(chromosome)

positionList <- split(position, chromosome)

positionList <- positionList[match(uchrom, names(positionList))]

arm <- list()

for(i in seq(along=uchrom)){

arm[[i]] <- as.integer(ifelse(positionList[[i]] <= chrAnn[uchrom[i],

"centromereStart"], 0, 1))

}

arm <- unlist(arm)

if(length(chromosome)==1) chromosome <- rep(chromosome, length(position))

splitby <- factor(cumsum(c(1, diff(x) != 0 | diff(arm) != 0)))

indices <- split(1:length(x), splitby)

len <- sapply(indices, length)

S <- states[sapply(split(x, splitby), unique)]

pos <- t(sapply(split(position, splitby), range))

size <- apply(t(sapply(split(position, splitby), range)), 1, diff)

chr <- sapply(split(chromosome, splitby), unique)

breaks <- data.frame(matrix(NA, length(chr), 7))

colnames(breaks) <- c("sample", "chr", "start", "end", "nbases", "nprobes", "state")

breaks$sample <- rep(sample, length(chr))

breaks$chr <- chr

breaks$start <- pos[, 1]

breaks$end <- pos[, 2]

breaks$nbases <- size

breaks$nprobes <- len

breaks$state <- S

if(!missing(lik1) & !missing(lik2)){

likdiff <- function(index, lik1, lik2, state){

state <- unique(state[index])

i <- range(index)

if(min(i) > 1) i[1] <- i[1]-1

if(max(x) < nrow(lik1)) i[2] <- i[2]+1

##the more positive the better

d1 <- diff(lik1[i, state])

d2 <- diff(lik2[i, "N"])

LR <- d1-d2

return(LR)

}

LR <- as.numeric(sapply(indices, likdiff, lik1=lik1, lik2=lik2, state=x))

}

breaks <- breaks[sapply(chr, length) == 1, ]

breaks$chr <- unlist(breaks$chr)

return(breaks)

}
